# Integrated assessment of CO_2_-ECBM potential in Jharia Coalfield, India

**DOI:** 10.1038/s41598-022-10574-5

**Published:** 2022-05-09

**Authors:** Mohammad Asif, Lei Wang, D. C. Panigrahi, Keka Ojha, Randy Hazlett

**Affiliations:** 1 School of Mining and Geosciences, Nazarbayev University, Kabanbay Batyr Ave 53, Nur-Sultan, 010000 Kazakhstan; 2 Present Address: College of Energy, Chengdu University of Technology, Chengdu, 610059 China; 3 IIT (ISM), Dhanbad, India; 4 Department of Petroleum Engineering, IIT (ISM), Dhanbad, India

**Keywords:** Environmental sciences, Energy science and technology, Engineering

## Abstract

Coalbed methane (CBM) production is effectively achieved by utilizing two processes, viz. primary and secondary recovery. In this paper, the primary recovery of CBM was studied using the adsorption isotherm while CO_2_-ECBM process for the secondary recovery was simulated with realistic parameters. The adsorption isotherm for CH_4_ was drawn up to the pressure of 1200 psi for four coal samples and Langmuir isotherm curves for both CH_4_ and CO_2_ was measured for one sample up to 2000 psi. The adsorption isotherm of four samples was further utilized for finding the primary recovery factor of methane, showing that the average primary recovery is ~ 54% with the highest recovery factor of ~ 76% for one sample. Hence, CO_2_-ECBM process could be further implemented to enhance gas recovery. Then, a 3D heterogeneous coalbed model at a depth of 3219 ft was constructed using the COMET3 simulator to demonstrate the potential of CO_2_-ECBM recovery technique. A concept of break-even time was introduced in this study for the comprehension of CO_2_-ECBM process. It is found that coalbed reservoirs may opt to implement this technology with economically sound recovery.

## Introduction

One of the critical challenges today is anthropogenic climate change due to global warming or rising earth temperature, of which a major contributor is the accumulated greenhouse gases, e.g., CO_2_, in the earth's atmosphere beyond the pre-industrial level. Global warming is affecting our planet via more extreme weather events as more heat is being trapped by greenhouse gases. There is an urgent demand to remove 1000 gigatons of CO_2_ from the earth's atmosphere by deploying more Carbon dioxide removal (CDR) techniques till 2100 to keep the warming below 1.5 °C^1^. As suggested by the world bank report, the world needs to adapt a systematic methodology to counterpoise 53 gigatons of greenhouse gas emitted each year^1^.

CO_2_ sequestration in geological formations, e.g. basalt formation, saline aquifers, unmineable coal seams, depleted coal and oil reservoir, is one of the effective techniques to meet the current challenges of growing greenhouse gases in the atmosphere. CO_2_ sequestration in unmineable coal seams is two-way advantageous for the future generation. It not only meets with the current challenges of greenhouse gas removal from the earth's atmosphere but also offsets the growing demand for energy through enhanced coalbed methane (ECBM) recovery. More and more research efforts have been devoted to developing the CO_2_-ECBM technology in the unmineable coal seams in recent years^2–8^. The first commercial ECBM field trial was performed in Allison unit, San Juan basin in 1995 and ~ 5 Bcf of CO_2_ had been injected with $2/Mcf profit in the CO_2_-ECBM project^9^. Others major ECBM pilot project includes Black Warrior basin, Powder river basin, Fenn-Big Valley (Alberta, Canada), Yuabri project (Hokkaido, Japan), China (Quinshui Basin (2004; 2010; 2013–15), APP ECBM project (2011–12)), etc.^3,4,10^.

Reservoir modeling is a methodology that incorporates various parameters related to geology, geochemical and petrophysics to replicate and predict the actual reservoir behaviour^6,11^. Like conventional reservoir modeling, CBM reservoir modeling is to aid in extracting the gas more efficiently, by predicting the reservoir performance with various possible technologies at different operating conditions and selecting the optimum conditions^12^. One of the main approaches to model CBM reservoir uses a non-equilibrium formulation in which sorption is pressure and time dependent and diffusion of gas in the coal matrix is the rate governing step. Various models have been put forth to understand the complex diffusion mechanism in coal, e.g. steady, pseudo-steady and unsteady state models^13–16^. The pseudo-steady state model considers matrix geometry and time, while the steady-state considers gas concentration as the primary controlling variable for calculating the diffusion process^17,18^. The unsteady state model is the most rigorous as it contemplates the gas concentration gradient^19^. Researchers have used various simulation software to model the CBM reservoir such as COMET3, ECLIPSE, COMSOL and SIMED-Win^20–24^. The COMET3 software used in this study, is a non-equilibrium pseudo-steady state simulator based on the dual porosity model^14,25^, which can incorporate vertical injection wells interrupting multiple seams, reservoir dip, stress-induced changes in permeability and porosity, and gravity segregation of gas–water system^20,23,26^.

Perera et al. (2012) utilized the COMET3 simulator for the numerical Simulation of CO_2_-ECBM reservoir^27^. Vishal et al.^20,23,24^ recently did a series of modeling studies for Indian Coalfields using COMET3 simulator. This paper discusses the primary CBM and CO_2_-ECBM recovery processes of Jharia coalfield. The primary recovery of the methane was comprehended with the help of adsorption isotherm. Numerical modeling was utilized for the secondary recovery of methane from Jharia coalfield. Simulation of CO_2_-ECBM process was done for ten years for Jharia coalfield. The breakthrough and break-even time, water and gas production rates for the ten years were extensively studied in this paper.

## Material and methods

### Sample collection preparation

**Figure 1:**
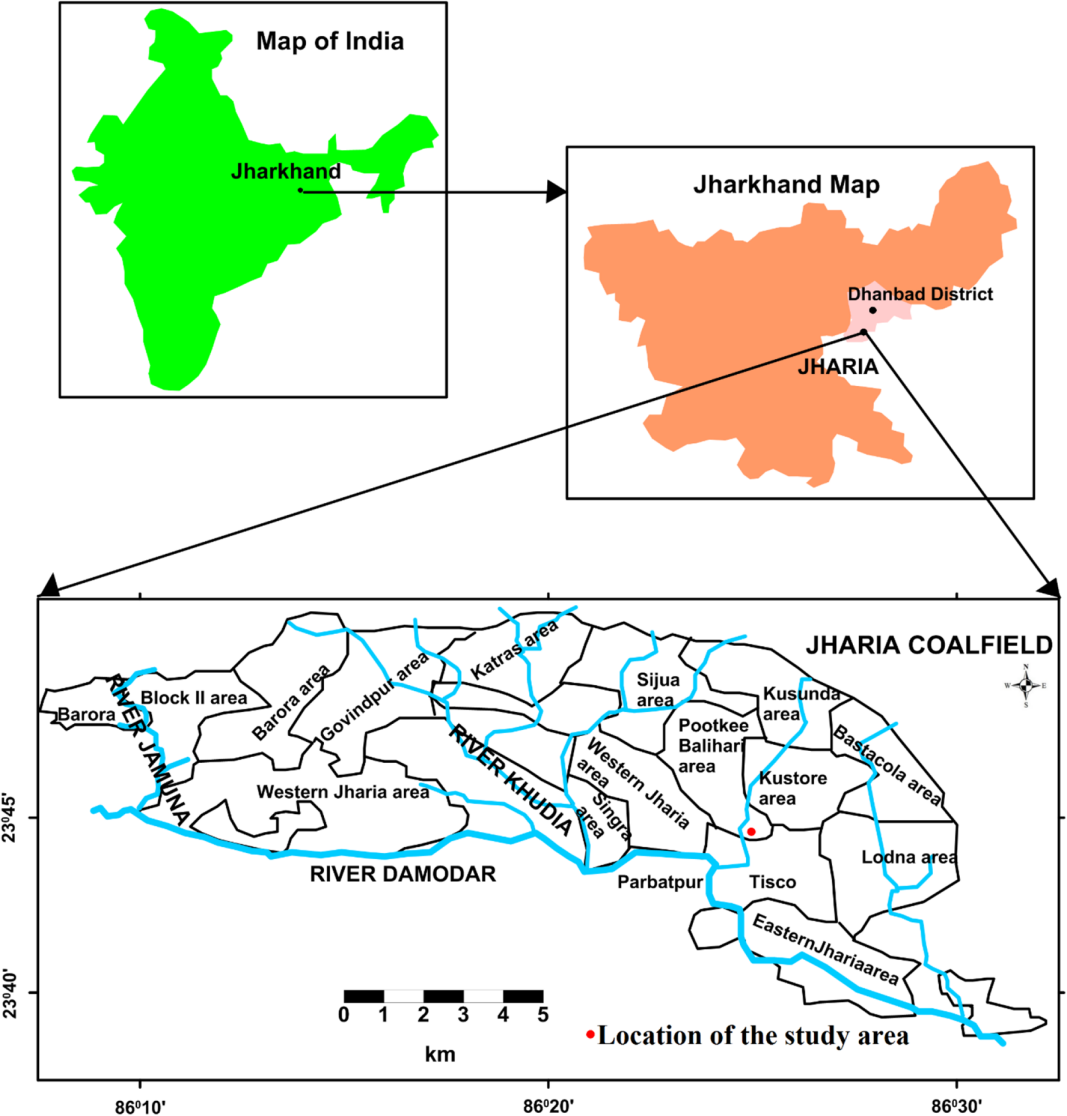
Location of coal samples in Jharia coalfield (Modified after Panigrahy et. al, 2015^29^ and plotted using Golden Software Surfer 11.0.642).

Four coal samples have been retrieved from the exploratory borehole of Jharia coalfield. The sickle-shaped Jharia coalfield was formed during the Permian age comprasing with Talchir, Barren measures, Barakar and Raniganj formation. Jharia coalfield is one of the main coalbed methane fields in India (Fig. 1). Barakar formation is the primary coal-bearing formation in the Jahria coalfield, and near about 18 coal seams that are mainly low to medium volatile bituminous coal present in this formation^28^. Nevertheless, high volatile bituminous coal is also revealed in some places. Jharia coalfield contains an enormous amount of methane for potential commercial CBM production.

Soon after retrieving the samples from the borehole, they were sealed in the desorption canister for measuring the gas content. After taking out the samples from gas canister, samples were wrecked in half-inch pieces and mechanically broken using a jaw crusher. Samples were then brought to a hammer mill for the reduction in size down to the mesh size of 8 ASTM (~ 2.38 mm). A series of experimental steps were used to obtain the desired particle sizes for various analyses. The perquisite samples were ground and sieved to a mesh size of 60/70 to obtain an average particle diameter of 230 µm^30^. Samples for the proximate, ultimate and adsorption analysis were prepared according to the same mesh size, while for petrographic analysis, polished specimen was made using 20 mesh coal sample. The lithology of coal samples confirms that it belongs to shaly coal to bright coal. The details of the samples are shown in Table 1.

**Table 1:** The description of the coal samples.

No	Sample ID	Depth Interval (m)	Mean depth (m)	Lithology
1	GC#1	831.3–831.8	831.55	Bright coal
2	GC#5	898.6–899.1	898.85	Bright coal
3	GC#7	937.6–938.1	937.85	Shaly coal
4	GC#14	981–981.5	981.25	Bright coal

### The gas content of the coal samples

**Figure 2:**
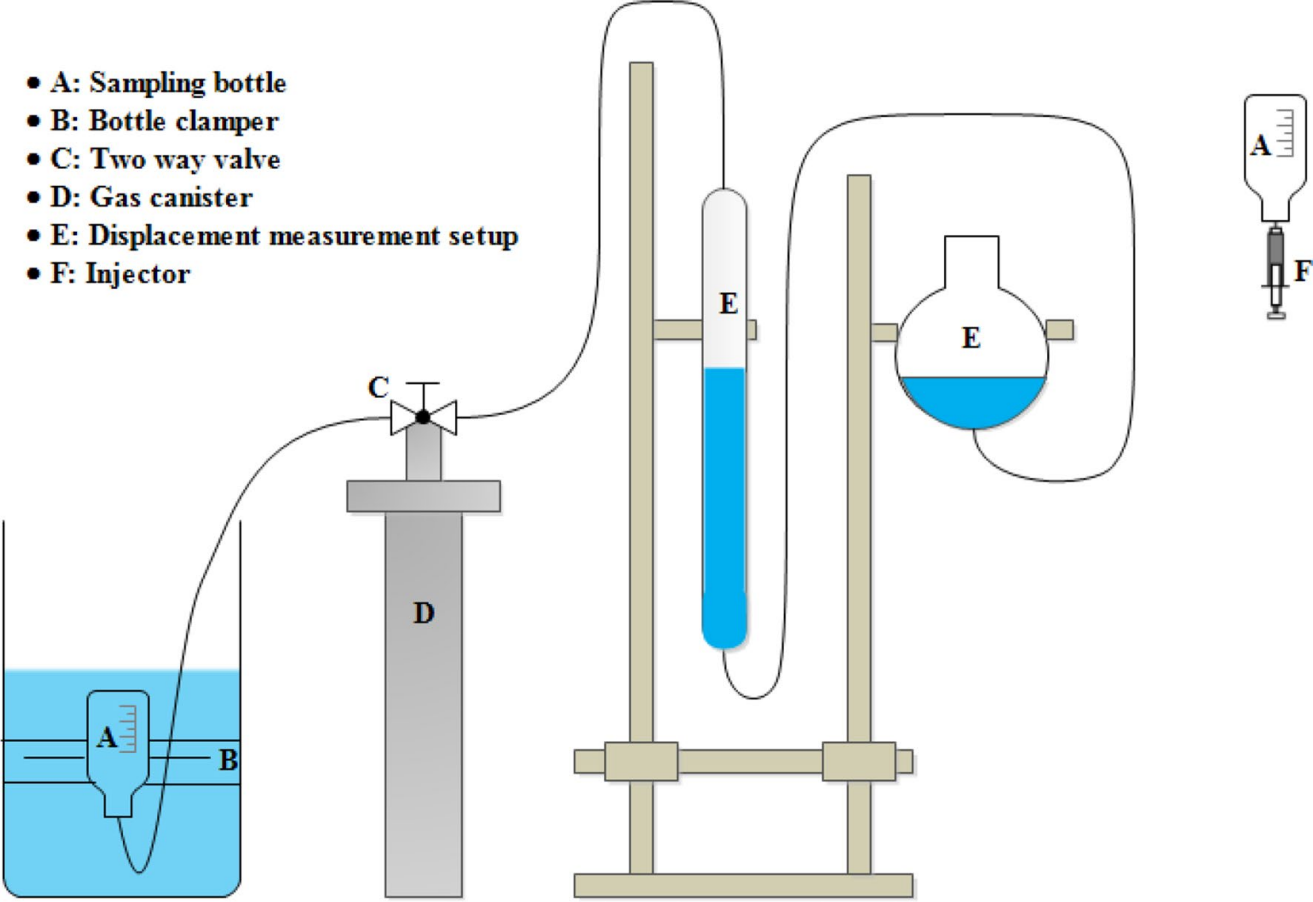
Set up for gas content measurement.

The gas content of the coal samples was analyzed using the United States Bureau of Mines (USBM) direct method^31,32^. The 50 cm freshly drilled borehole sample was sealed into the canister, and desorbed gas content was measured using the water displacement method. The actual gas content (G) adds the lost, desorbed and residual gas content as revealed in Eq. (1)^33^.

1 $$G(cc/g) = \frac{{Q_{1} + Q_{2} + Q_{3} }}{w}$$

where Q_1_, Q_2_ and Q_3_ are the lost, desorbed and residual gas in cc, while w (g) is the borehole sample weight. Figure 2 described the combined experimental set-up for collection and measurement of desorbed gas in field as well as in laboratory. The section including ABCD is used in the field for determination of desorbed gas immediately after retrieval of core from the well. The core was kept inside the canister D and the desorbed gas from the core was stored in graduated sample bottle A (fixed with clamp), dipped in the saline water through a two-way valve C. Once the system is brought to the laboratory, the valve is opened to the burette E through valve C and finally stored in collector “E”. For laboratory analysis, the gas samples can alsobe connected to sample collector A.

The coalbed gas content by dry and ash-free basis (*G*_*daf*_) is defined by Eq. (2). The gas/vapor including moisture is adsorbed on the active surface area of the coal. In presence of ash, the active surface is reduced. Methane and moisture are competitive to the adsorption sites. Hence, the CBM gas content is decreased with the increament in ash content and moisture content.

2 $$G_{daf} (cc/g) = \frac{G}{1 - a - m}$$

where a and m are the ash and moisture fraction of coal, respectively.

### Sample characterization

Proximate, ultimate and petrographic analyses were utilized for the characterization of the coal samples. The proximate analyses of the collected samples were carried out using the Advanced Research Instrument-Proximate Analyzer (APA-2) by following the standardASTM D3172-07a (2007). Ultimate analysis of coal was conducted using Elementar Vario MICRO cube (EL III CHNS analyzer) by following the standard ASTM D3176-89 (2002). ASTM standard (ASTM D2798-18, 2018) was utilized to measure the vitrinite reflectance of coal samples.

### Adsorption isotherm

**Figure 3:**
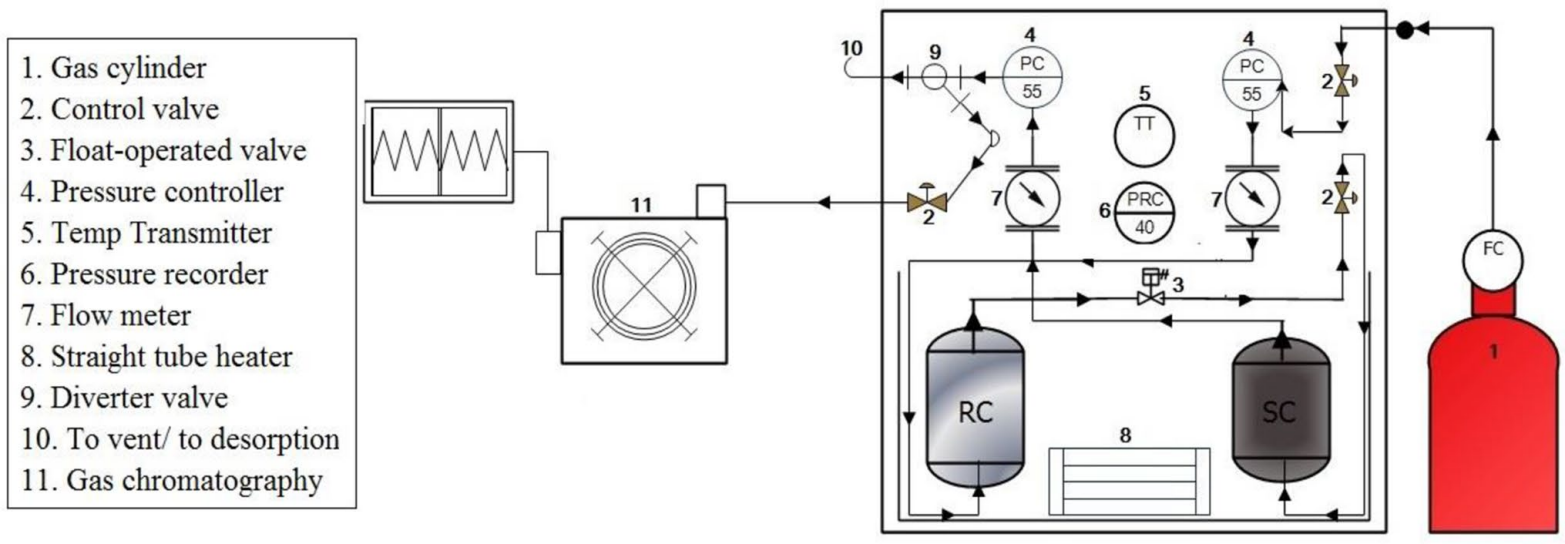
The schematic diagram for the gas adsorption^30^.

Adsorption isotherm for the coal were obtained using the indigenous setup based on the manometric method. This experiment was carried out at the reservoir condition for correctly depicting the methane adsorption capacity of coal. The schematic diagram for the experiment is shown in Fig. 3. The volume of gas adsorbed at different pressure and temperature conditions were measured using this apparatus. The details of the schematics diagram are described in another published literature^30^.

The Langmuir equation was used to calculate the volume of pure gas adsorbed (V) at a particular pressure (P).

3 $$V = \frac{{PV_{L} }}{{P + P_{L} }}$$

where *P*_*L*_ and *V*_*L*_ are the Langmuir pressure and volume, respectively.

## Numerical modeling of CO_2_ ECBM process

Modeling of CO_2_-ECBM process was carried out after studying the coal samples' geological and geochemical parameters. The coalbed model includes heterogeneity and anisotropy in coal, dual-porosity (microspores and macropores), gas diffusion by Fick's second law, Darcy's law for flow in cleats, preferential adsorption of CO_2_ over CH_4_, shrinkage and swelling of coal, permeability changes due to CO_2_ injection, and mixture adsorption using extended Langmuir model. COMET3 simulator was used for CO_2_-ECBM modeling of Jharia coalfield. The modeling was done for 3650 days, i.e. CBM was substituted by CO_2_ for ten years.

### Governing equations for the modeling study

The governing equations in the COMET3 simulator are described below^20,34^:

Mass conservation equation for gas:

4 $$\nabla .\left[ {b_{g} M_{g} \left( {\nabla p_{g} + \gamma_{g} \nabla Z} \right) + R_{sw} b_{w} M_{w} \left( {\nabla p_{w} + \gamma_{w} \nabla Z} \right)} \right]_{f} + q_{m} + q_{g} = \left( \frac{d}{dt} \right)\left( {\phi b_{g} S_{g} + R_{sw} \phi b_{w} S_{w} } \right)_{f}$$

Mass conservation equation for water:

5 $$\nabla .\left[ {b_{w} M_{w} \left( {\nabla p_{w} + \gamma_{w} \nabla Z} \right)} \right]_{f} + q_{w} = \frac{d}{dt}\left( {\phi b_{w} S_{w} } \right)_{f}$$

where *b*_*n*_* (n* = *g or w)* is the bulking factor for gas and water, $\gamma_{n}$ is the gradient, *R*_*sw*_ is the solubility of the gas in water, $\phi$ is the porosity of coal, *Z* is the elevation, *q*_*n*_ is the well sources term for gas and water, *q*_*m*_ is the flow of gas in the matrix and *M*_*n*_ is the mobility of gas and water.

The adsorption–desorption of the mixture of CO_2_/CH_4_ was described by the extended Langmuir equation, which gives the amount of a particular gas adsorbed (*V*_*i*_)^35^.

6 $$V_{i} (scf/ton) = V_{Li} \frac{{\frac{{Py_{i} }}{{P_{Li} }}}}{{1 + \sum\limits_{j = 1}^{{n_{c} }} {\frac{{Py_{j} }}{{P_{Lj} }}} }}$$

where j indicates gas components (*CH*_*4*_ or *CO*_*2*_), *n*_*c*_ is number of gas components, *V*_*Li*_ and *P*_*Li*_ are the Langmuir volume and pressure of component *i*, *y*_*i*_ is free gas mole fraction of component *i*, and *P* is pressure, psia.

The flow of gas in the matrix is described with Fick's second law of diffusion:

7 $$q_{{m_{i} }} = \frac{{V_{m} }}{{\tau_{i} }}\left[ {C_{i} - C_{i} (P_{i} )} \right];i = 1,2$$

where *q*_*m*_ is the flow of particular gas, *V*_*m*_ is the element volume of the matrix, sorption time is $\tau_{i}$, and *C*_*i*_ is the concentration of gas component *i.*

The porosity and permeability equations of the gas used are the Advance Resource International (ARI) models^20^:

8 $$\phi = \phi_{0} \left[ {1 + c_{p} \left( {P - P_{0} } \right)} \right] - c_{m} \left( {1 - \phi_{0} } \right)\left( {\frac{{\Delta P_{0} }}{{\Delta C_{0} }}} \right)\left( {C - C_{0} } \right)$$

9 $$\frac{k}{{k_{0} }} = \left( {\frac{\phi }{{\phi_{0} }}} \right)^{n}$$

where *C*_*p*_ and *C*_*m*_ are the pore and matrix shrinkage compressibility respectively; $\phi$ and $\phi_{0}$ are the coal porosity and initial coal porosity respectively, *P* and *P*_*0*_ are the reservoir pressure and initial reservoir pressure, *C* and *C*_*0*_ are the reservoir concentration and initial reservoir concentration respectively, *k* is the permeability and *k*_0_ is the initial permeability. Equation (9) is an empirical correlation based on the Kozeny-Carman equation with the exponent fitted to relate the permeability with porosity, which are measured from experiments. In our studies, we did not measure these values, the value of n in general ranges from 2 to 4.

### Assumptions made for modeling

1. The coalbed has no dipping angle with constant thickness
2. The coalbed reservoir temperature is uniform and constant
3. Darcy's law is followed for describing the flow through cleats and fractures
4. The Fick's second law gives the diffusion in the coal matrix
5. The pseudo-steady state condition exists within a finite-difference grid block
6. The grid or finite difference blocks of the model is homogeneous or sorption time is constant within a block

### The coalbed block model

**Figure 4:**
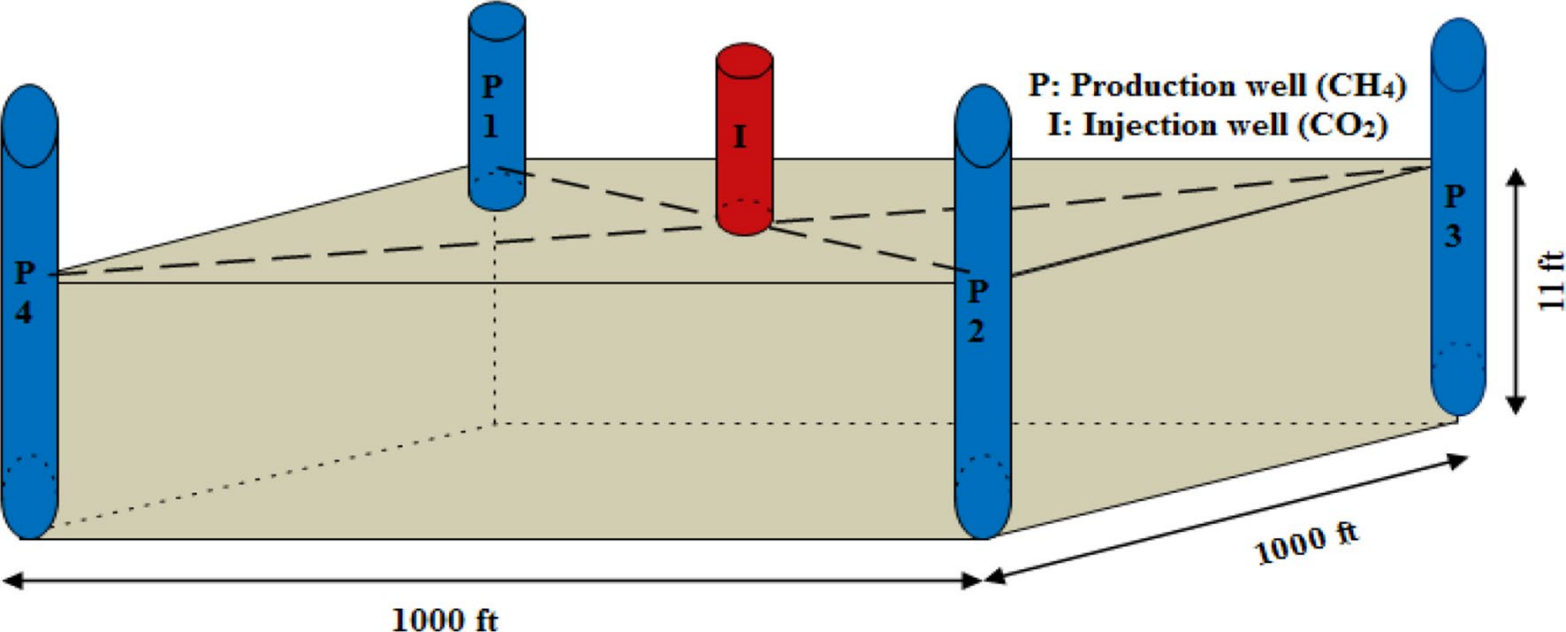
Coalbed block for CO_2_-ECBM simulation.

The dimension of the coalbed block was chosen as 1000 ft × 1000 ft × 11 ft at a depth of 3219 ft with four production wells and one injection well. The production wells were selected as diagonally opposite with injection well at the center (Fig. 4). The main parameters are the Langmuir constants for both components, relative permeability curve, reservoir temperature, gas content, wellbore dimension, pore pressure, porosity, permeability, pore and matrix compressibility of coal.

The model with two phases (gas–water), two components (CO_2_–CH_4_), and dual porosity (micro and macropores) was chosen to simulate the CH_4_ production by CO_2_ injection. The total coal block area is 23 acres. The coalbed model was divided into 20 grids of 50 ft each in x and y direction while 1 grid of 11 ft in z-direction.

The well diameter of the injection and production wells were chosen as 8 inches with a tubing diameter of 5 inches. CO_2_ was injected at the bottom of the model. Parameters for the model are tabulated in Table 2.

**Table 2:** Input parameters for modeling.

Input parameters		Values
Reservoir Parameters		
Depth (ft)		3219
Thickness (ft)		11.1
Matrix permeability (mD)		1.5
Cleat permeability (mD)		6.09
Matrix porosity (%)		0.745
Cleat porosity (%)		0.18
Reservoir temperature (ͦ F)		139
Reservoir pressure (psi)		135
Gas content (scf/t)		434.74
Pore compressibility (psi^-1^)		1.213E-03
Matrix compressibility (psi^-1^)		1.767E-07
V_L_–CH_4_ (scf/t)		1428.57
P_L_–CH_4_ (psi)		961.43
V_L_–CO_2_ (scf/t)		3142.85
P_L_–CO_2_ (psi)		1282.4
Fluid parameters		
Gas gravity		0.58
Water viscosity (cp)		0.8
Water formation volume factor (RB/STB)		1
Well parameters		
Casing ID (inch)		8
`Tubing outer diameter (inch)		5
Skin factor	Production well	0
	Injection well	−2.5

## Results and discussions

### Gas content of the coal samples

The gas content of the coal samples is shown in Table 3. The maximum coalbed gas content was found as 9.98 cc/g for sample GC #14. The gas content on the *daf* basis was also calculated.

**Table 3:** Gas content of the coal samples.

Sample	*w, g*	*Q*_*1*_*, cc*	*Q*_*2*_*, cc*	*Q*_*3*_*, cc*	*Q, cc*	*G, cc/g*	*G*_*daf*_*, cc/g*
GC#1	1130	116	688.41	747.48	1551.89	1.37	1.99
GC#5	1180	358	1602	2419.37	4379.37	3.71	5.45
GC#7	1350	380	3635.03	549.27	4564.3	3.38	4.77
GC#14	1130	437	10,352.14	492.6	11,281.74	9.98	13.57

### Characterization of coal samples

Samples were characterized based on proximate analysis, ultimate analysis, and vitrinite reflectance. As per the standard ASTM D388-18 (2018) for classification of rank and Stach's coal rank chart, coal samples belong to low volatile to medium volatile bituminous coal^36^. The characteristics of coal samples are shown in Table 4.

**Table 4:** Characteristics of coal samples.

Component	G.C. #1	G.C. #5	G.C. #7	G.C. #14
**Proximate analysis (%, adb)**				
A	30.41	30.12	31.17	28.61
M	0.78	0.75	0.79	0.68
VM	18.85	13.74	14.75	14.59
FC	49.96	55.39	53.29	56.12
*FC*^*daf*^	72.61	80.12	78.32	79.37
*VM*^*daf*^	27.39	19.88	21.68	20.63
*Fuel Ratio (FC/VM)*	4.03	3.61	3.85	4
**Ultimate analysis (%, adb)**				
C	67.86	65.87	65.64	78.26
H	3.35	3.54	3.34	3.18
N	2.08	2.14	2.41	1.97
S	0.79	3.64	0.68	1.29
O	25.92	24.81	27.93	15.3
**Vitrinite reflectance (R**_**o**_**, %)**				
*R*_*o*_* (%)*	1.37	1.37	1.42	1.46

### Adsorption isotherm and primary recovery

**Figure 5:**
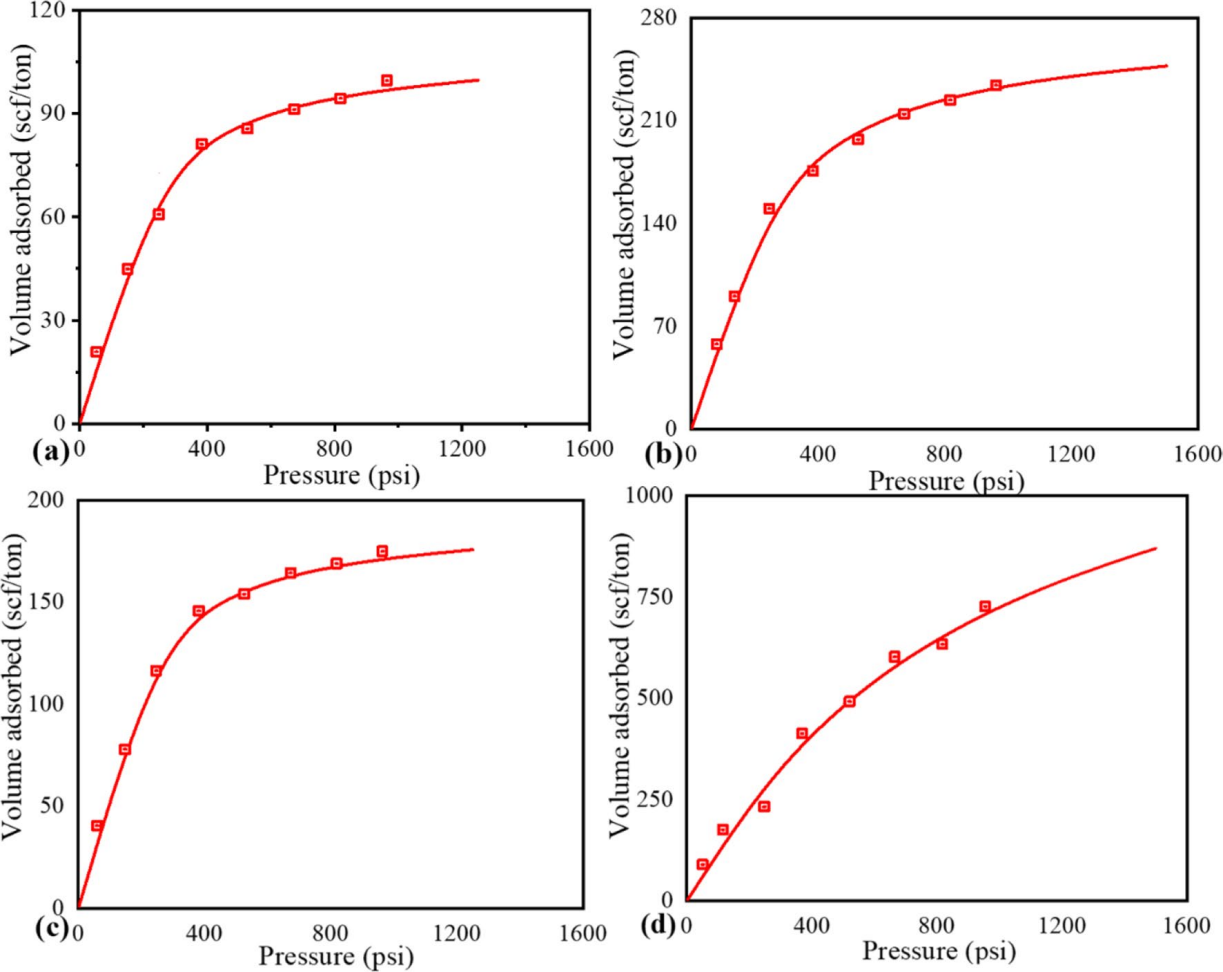
The CBM adsorption isotherms of the coal samples: (**a**) G.C. #1 (**b**) G.C. #5 (**c**) G.C. #7 (**d**) G.C. #14.

The CBM adsorption isotherms for the four samples and their respective Langmuir parameters are shown in Fig. 5a–d. The Langmuir constants obtained through regression analysis by fitting the adsorption data with the Langmuir equation are shown in Table 5. The isotherm curves of the samples belong to Type I adsorption isotherm curve^37^.

**Table 5:** Langmuir constants of the coal samples.

Sample number	Sample ID	*T*_*r*_* , °C*	*P*_*L*_* , psi*	*V*_*L*_* , scf/ton*
1	GC#1	54.95	128.32	109.89
2	GC#5	56.97	186.56	277.78
3	GC#7	58.14	118.25	192.31
4	GC#14	59.44	961.43	1428.57

*Tr* Reservoir temperature.

The primary recovery of methane was found using the analogy described in our previous publication^31^. The primary recovery of methane was analyzed using the adsorption isotherm with the help of critical desorption pressure and gas content. The dewatering process also plays an important role in the primary recovery of methane^31^. The CBM recovery factors from these samples were calculated using Eq. (10) and are shown in Table 6.

10 $$R_{c} = \frac{{V_{cdp} - V_{ab} }}{{V_{cdp} }} \times 100\%$$

**Table 6:** The primary recovery factors of methane.

Sample ID	Gas Content (scf/ton)	*P*_*r*_* (psi)*	*P*_*cdp*_* (psi)*	*P*_*ab*_* (psi)*	Under saturation	*V*_*cdp*_* (scf/ton)*	*V*_*ab*_* (scf/ton)*	*R*_*c*_*(%)*
GC #1	63.75	1173.48	177.3	75	55	63.75	40.54	36
GC#5	174.6	1268.46	315.69	75	39	174.6	79.65	54
GC#7	152.82	1323.49	457.61	75	16	152.82	74.64	51
GC#14	434.74	1384.74	420.57	75	94	434.74	103.38	76

*P*_*r*_   Reservoir pressure*, P*_*cdp*_   Critical desorption pressure*, P*_*ab*_   Abandoned Pressure*, V*_*cdp*_   Gas content at* P*_*cdp*_*, V*_*ab*_   Gas content at *P*_*ab*_*, R*_*c*_   Recovery factor*.*

### Modeling results of the CO_2_-ECBM process

**Figure 6:**
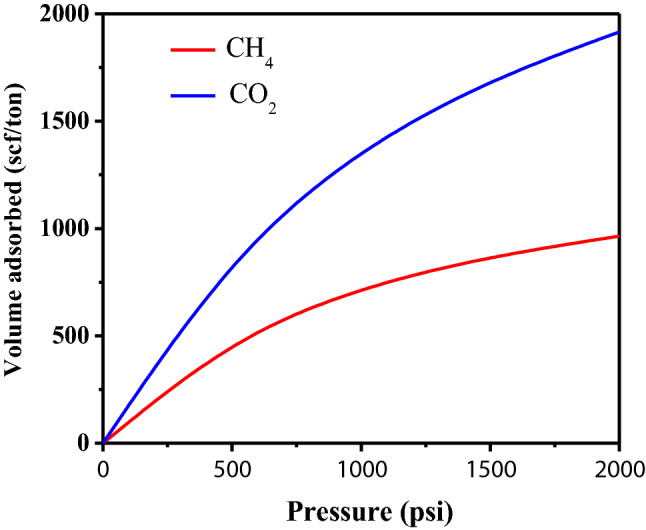
Langmuir curves for both CH_4_ and CO_2_.

The Langmuir isotherm curves obtained for CH_4_ and CO_2_ are shown in Fig. 6, and these curves were used in the simulation.

**Figure 7:**
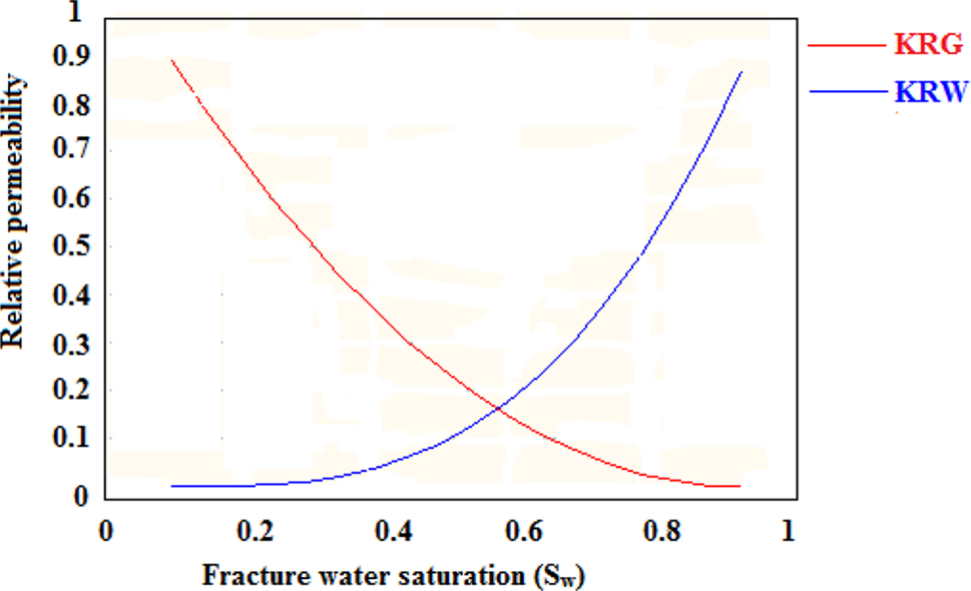
Relative permeability curve for the gas and water.

Similarly, the water and gas relative permeability curves were also one of the essential parameters for simulating the CO_2_-ECBM process. Relative permeability controls the production performance of the CBM reservoir, and determination of realistic curves is challenging^38,39^. The relative permeability curves for gas and water at varying saturation levels used here are drawn using the Corey correlation based on the Corey capillary pressure model^40^, as shown in Fig. 7.

#### Matrix CO_2_ and CH_4_ concentration

**Figure 8:**
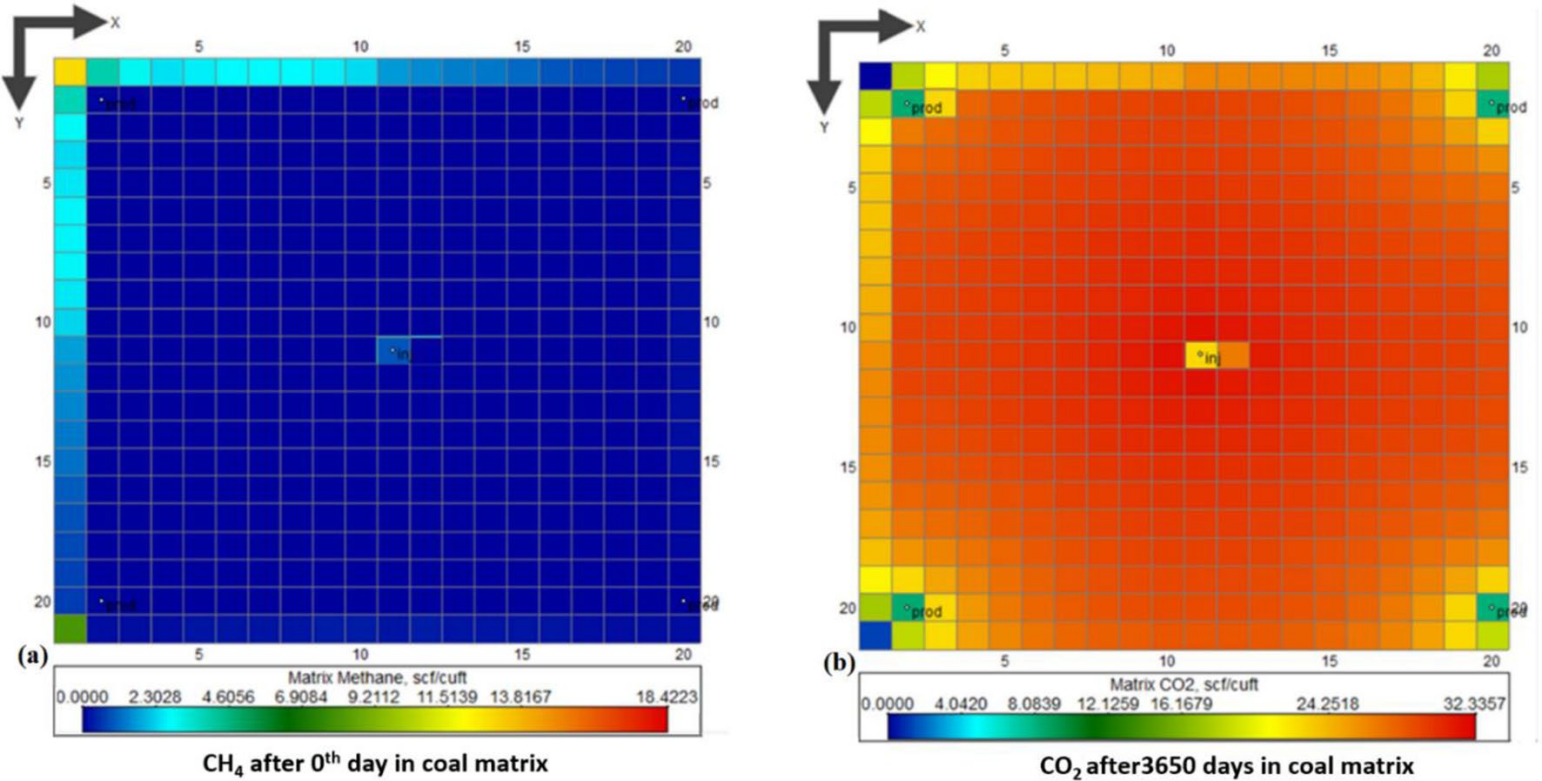
CH_4_–CO_2_ matrix concentration after 0 day and 3650 days respectively: (**a**) CH_4_ (**b**) CO_2_.

CO_2_ injected in coalbed dispersed from the center to the production wells at the corner of the block. Figure 8 reveals the CH_4_ and CO_2_ concentrations in coalbed grids with anisotropy and heterogeneity.

Figure 8a shows the methane concentration at 0 day, i.e., before the injection, there is only methane in the coal matrix, and slowly it desorbs due to the displacement of CO_2_. After 3650 days, nearly all methane would be desorbed from the coal matrix, and high concentration of CO_2_ is shown in the coal matrix of the selected coal block, as shown in Fig. 8b. The maximum displacement rate of methane is achieved after 87 days of injection, i.e., 452 MSCFD.

#### CH_4_ and CO_2_ production and injection analysis

**Figure 9:**
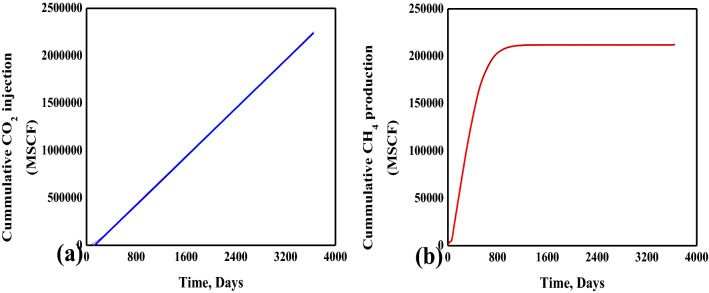
CO_2_ injection and CH_4_ production curves.

Both CO_2_ injection and CH_4_ production started at the same time. Although the production wells are placed equidistant from the injection well, the anisotropic permeability of the coal results in the rough start of the CH_4_ production of from the production wells^41^. The cumulative CO_2_ injection in Fig. 9a is linear because injection rate was fixed. Totally 2.25 BSCF of CO_2_ is required for injection till ten years. The cumulative CH_4_ production shown in Fig. 9b indicates that the maximum cumulative production of CH_4_ is achieved after ~ 700 days, due to the breakthrough of CO_2_ in the production wells.

**Figure 10:**
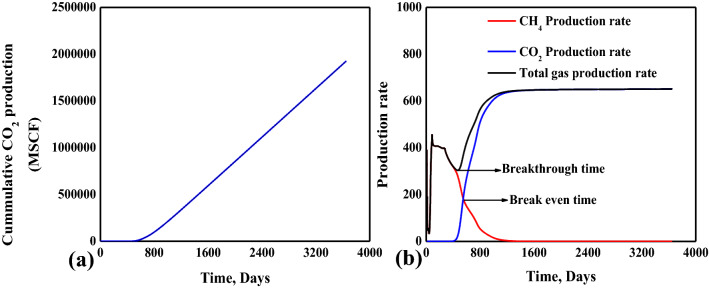
Breakthrough time and production curves.

The very early breakthrough of the CO_2_ is after ~ 274 days, as shown in Fig. 10a. After breakthrough, the CO_2_ production linearly increases up to 3650 days. The breakthrough time was analyzed with methane and gas production rates, as shown in Fig. 10b. The point where the methane production rate and total gas production rate curves bifurcate would be the breakthrough time. The CO_2_ production rate after ~ 554 days exceeds the CH_4_ production rate. This point is designated as break-even time. After this time, the production of CH_4_ should be critically analyzed as separation cost of CO_2_ would be high. The shrinkage and swelling effect is also one of the leading causes of the depletion in CH_4_ production rate^42,43^.

#### Total water and gas production

**Figure 11:**
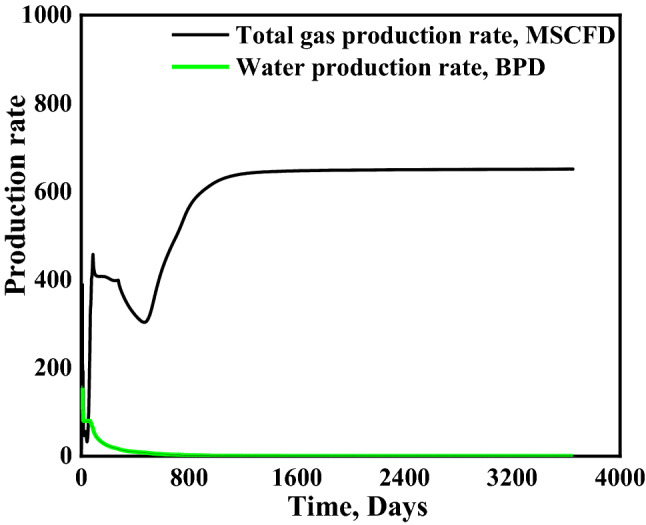
The gas and water production rates.

Dewatering is a crucial stage for CBM production in coalbed methane reservoirs. Some of the wells could produce water up to 2–3 years^44,45^. The dewatering is required to reach the critical desorption pressure, and more dewatering means that the methane recovery factor would be lower ^9,46^. Thus, water production for the coalbed methane reservoiris critical for the CBM project economics. In this simulation, water production rate remains high (~ 50 BPD) for the first ~ 80 days and starts decreasing drastically (Fig. 11). The water production rate drops to ~ 10 BPD at the end of the first year, when total water production is around 14,000 BBLS. However, after ten years, i.e. at the end of this study, the water production rate of the water becomes almost 0 (~ 0.2 BPD), and the total water production is estimated to be ~ 18,000 BPD. It can be observed that after 55 days the total gas production rate surpasses the water production rate.

## Conclusions

Primary recovery of coalbed methane requires dewatering to reduce the reservoir pressure to desorb methane gas from the coal matrix. CO_2_ injection displaces methane and reduces the partial pressure of methane, and its adsorption on coal surface further removes adsorbed methane. The following conclusions can be drawn from this study:

1. Primary recovery on coal samples shows that more than 50% of methane can be extracted from the coalbed except for one sample.
2. Simulation case study showed that five years are required to desorb all the methane through CO_2_-ECBM, after that methane production rate drops to zero.
3. The maximum methane production rate can be achieved after 87 days, which is ~ 452 MSCFD. Meanwhile, the cumulative methane production reaches 15,308 MSCF. The CO_2_ amount in the cumulative gas production becomes noticeable after one-year injection, i.e. ~ 1 MSCF. Nevertheless, the production rate of CO_2_ at this point is 0.1 MSCFD.
4. Water production in the coalbed methane reservoir plays a crucial role in determining the overall project economy. Cumulative water production in this numerical case is continuous from the beginning and becomes constant (~ 17,000 BBLS) after four years of injection.

This integrated experimental and numerical study is valid and helpful for future implementation of the value adding CO_2_-ECBM process in the Jharia Coalfield, India. Before realizing this technology in the coalfield, more comparative studies on well types and patterns^47^, economic analysis and feasibility study must be carried out.

## List of symbols

*Q*_*1*_: Lost gas
*Q*_*2*_: Residual gas
*Q*_*3*_: Lost gas
*Q*: Total gas
*G*: Gas content (as received basis)
*G*_*daf*_: Gas content (dry-ash free basis)
*V*_*L*_: Langmuir volume, Scf/ton
*P*_*L*_: Langmuir Pressure, psi
*b*_*g*_: Bulking factor for gas
*b*_*w*_: Bulking factor for water
$\gamma_{g}$: Gradient for gas
$\gamma_{w}$: Gradient for water
*R*_*sw*_: Solubility of the gas in water
*q*_*g*_: Well sources term for gas
*q*_*w*_: Well sources term for water
*q*_*m*_: Flow of gas in the matrix
$\phi$: Porosity of coal
*M*_*g*_: Mobility of gas
*M*_*w*_: Mobility of water
*A*: Ash content (%, adb)
*M*: Moisture content (%, adb)
*VM*: Volatile matter (%, adb)
*FC*: Fixed Carbon (%, adb)
*FC*^*daf*^: Fixed Carbon (%, daf)
*VM*^*daf*^: Volatile matter (%, daf)
*C*: Carbon content (%, adb)
*H*: Hydrogen content (%, adb)
*N*: Nitrogen content (%, adb)
*S*: Sulfur content (%, adb)
